# Diabetes Mellitus and Risk of Future Stroke: Evidence From CHARLS and Mendelian Randomization Analyses

**DOI:** 10.1002/brb3.70151

**Published:** 2024-11-17

**Authors:** Zetai Bai, Zheyi Wang, Mei Li, Deyuan Kong, Guanzhao Wu

**Affiliations:** ^1^ Department of Central Laboratory and Mitochondrial Medicine Laboratory, Qilu Hospital (Qingdao), Cheeloo College of Medicine Shandong University Qingdao China; ^2^ School of Clinical Medicine University of Health and Rehabilitation Sciences Qingdao Shandong China; ^3^ School of Clinical Medicine Shandong Second Medical University Weifang Shandong China

**Keywords:** diabetes mellitus, hemoglobin A1c, Mendelian randomization analysis, stroke, vascular diseases

## Abstract

**Objectives**: This study leveraged the China Health and Retirement Longitudinal Study (CHARLS) to explore the association between diabetes and stroke in middle‐aged and older adults in East Asia and assess the causality of this relationship using Mendelian randomization.

**Methods**: Data from the 2011–2020 CHARLS cohort identified individuals with diabetes at baseline. Stroke incidence was self‐reported through standardized questionnaires. Logistic regression and restricted cubic spline analysis examined the relationship between diabetes and stroke risk alongside nonlinear correlations between glucose levels and stroke. Mendelian randomization clarified the causal link and analyzed the mediating effect between diabetes and stroke using genetic methods.

**Results**: In the study population aged 45 and above, stroke incidence was 5.99% in normoglycemic, 6.82% in prediabetic, and 9.93% in diabetic individuals. Over 7 years, 473 strokes occurred. Diabetes was associated with a 1.35‐fold increased stroke risk compared to normoglycemia (OR = 1.35; 95% CI: 1.03–1.79). Subgroup analyses highlighted higher stroke risks in middle‐aged women, nonsmokers, and nondrinkers. Mendelian randomization supports a genetic causal relationship between diabetes and stroke. Diabetes may indirectly lead to stroke through the mediating effects of hypertension and high cholesterol.

**Conclusion**: The findings confirm a significant association and causal link between diabetes and stroke risk in an East Asian population. In addition, the results indicate that controlling blood glucose in prediabetic individuals reduces stroke risk, with no similar benefits in diabetes.

## Graphical abstract

The study establishes a significant association and causal link between diabetes and stroke risk in an East Asian population.

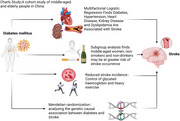

## 1 Introduction

Stroke remains the second leading cause of mortality globally, imposing a substantial burden on healthcare systems worldwide (Feigin et al. 2022; Naghavi et al. 2017; Roth et al. 2020). Defined as a rapid onset of brain dysfunction lasting more than 24 h or resulting in death, stroke typically arises from cerebral blood vessel rupture or obstruction, leading to compromised blood flow to the brain (Coupland et al. 2017; Sacco et al. 2013; Buck et al. 2021). Despite a declining age‐standardized mortality rate for stroke on a global scale, the incidence of stroke cases and associated fatalities continue to rise, particularly in aging populations like China's, exacerbating strain on the healthcare infrastructure (Wu et al. 2019; Peng et al. 2023; Tu et al. 2022).

Despite the various treatment options currently available for stroke, the primary focus remains on identifying and managing stroke risk factors to alleviate the growing stroke burden in China (Wang et al. 2024; Ran et al. 2024; He et al. 2023; Kan et al. 2023). Diabetes mellitus (DM), characterized by chronic hyperglycemia stemming from impaired glucose metabolism, stands out as a significant risk factor for vascular diseases, precipitating various complications within the vascular system, including endothelial dysfunction and atherosclerosis (Chawla, Chawla, and Jaggi 2016; Zhou et al. 2022; Maranta, Cianfanelli, and Cianflone 2020). Multiple mechanisms link diabetes to stroke, including vascular endothelial dysfunction, cardiac embolism, atherosclerosis, systemic inflammation, and capillary basement membrane thickening (Chawla, Chawla, and Jaggi 2016; Mosenzon et al. 2023; Maida et al. 2022; Bradley et al. 2022). While prior research underscores the heightened stroke risk associated with DM, a paucity of cohort studies focusing on middle‐aged and elderly Chinese individuals with diabetes exists despite their elevated stroke risk. These studies, characterized by smaller sample sizes and shorter follow‐up durations, underscore the need for further investigation (Hillen et al. 2003; Purroy et al. 2012; Hankey et al. 1998).

Simultaneously, the relationship between glycemic abnormalities and stroke incidence remains nebulous. Unlike fasting blood glucose (FBG), hemoglobin A1c (HbA1c) testing offers the advantage of assessing average blood glucose levels over the preceding 120 days (Joshi et al. 2024; Kaiafa et al. 2021; Lundholm et al. 2020), furnishing a more reliable indicator of abnormal blood sugar levels and mitigating the risk of stress‐induced hyperglycemia misdiagnosis (Roberts et al. 2021; Vedantam et al. 2022). Routine HbA1c testing may thus serve as a pivotal tool in identifying and optimizing blood sugar management, thereby attenuating stroke risk (Banerjee et al. 2012; Kovatchev 2017; Beck et al. 2019).

To delve deeper into the diabetes‐stroke nexus, we conducted a prospective cohort analysis leveraging data from the 2011–2020 China Health and Retirement Longitudinal Study (CHARLS). This investigation aimed to delineate stroke risk across diverse subgroups, including diabetes duration, gender, smoking and alcohol habits, and physical activity levels. In addition, a meta‐analysis employing Mendelian randomization (MR) across multiple databases affirmed a genetic causal association between diabetes and stroke.

## 2 Methods

### 2.1 Study Design and Populations

**FIGURE 1:**
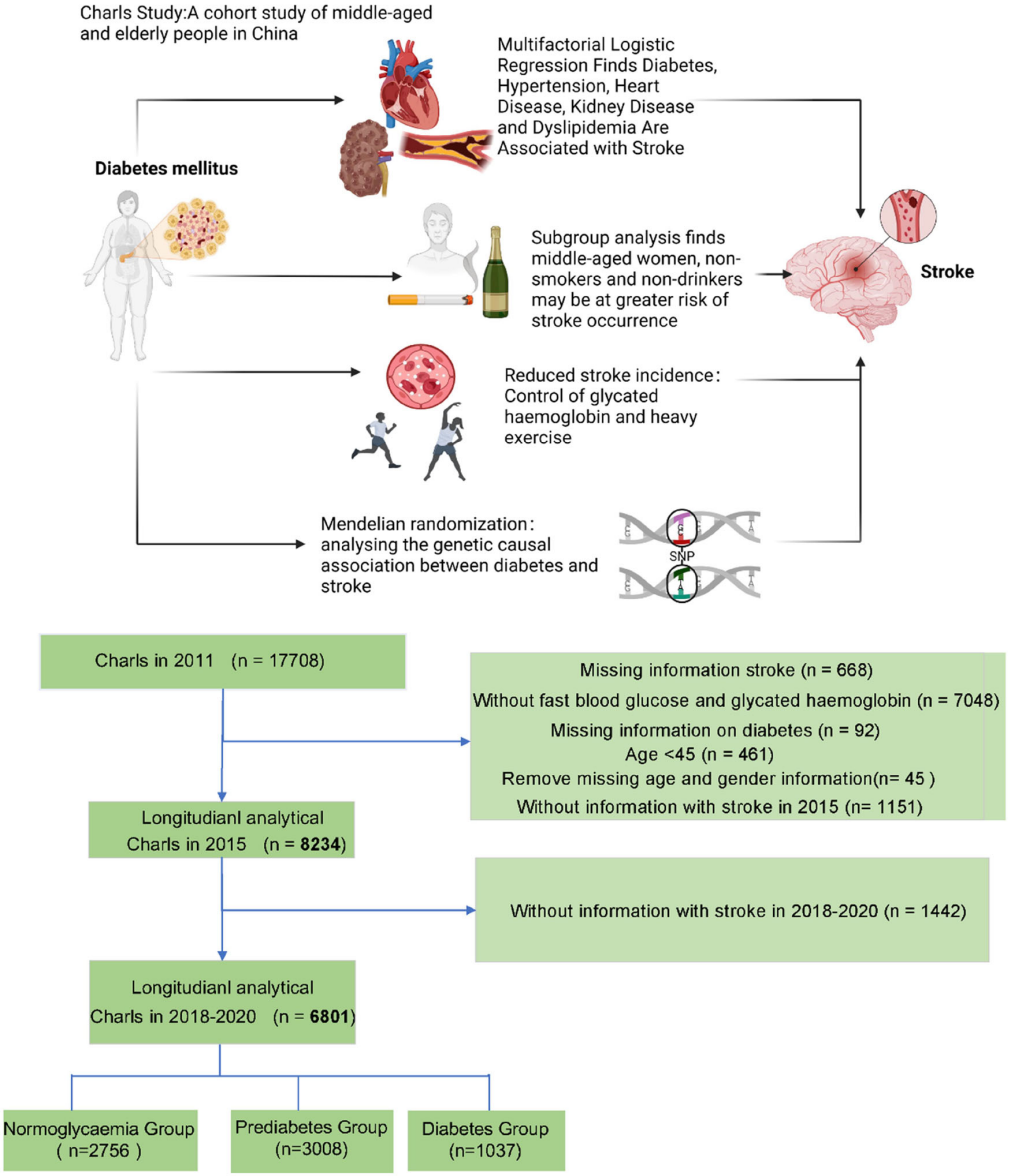
Participant selection process flowchart.

CHARLS constitutes an ongoing national cohort investigation leveraging data from the China Health and Retirement Longitudinal Study (CHARLS). The inaugural national baseline survey of the CHARLS database commenced in 2011, encompassing 17,708 participants drawn from 10,257 households. The current study involves a secondary analysis of CHARLS baseline and 2011–2020 follow‐up data. Rigorous standardized questionnaire surveys were administered to gather comprehensive sociodemographic, lifestyle, and health‐related data. Exclusionary criteria comprised individuals with a documented history of stroke predating 2011, those lacking complete FBG and HbA1c data, participants under the age of 45, individuals with missing gender and age information, and those devoid of stroke follow‐up records between 2015 and 2020, as well as individuals lacking diabetes information. Adhering to these criteria, a total of 6801 participants were recruited (Figure 1). The CHARLS study holds approval from the Institutional Review Board of Peking University, with written informed consent procured from all participants. Comprehensive data and detailed documentation are publicly accessible at https://charls.charlsdata.com/pages/data/111/zh‐cn.html, with requisite approval secured for utilization of the CHARLS database. Ethical approval for the study was obtained from the Biomedical Ethics Review Committee of Peking University (IRB00001052‐11015), and all participants provided written consent prior to inclusion.

### 2.2 Identification of Stroke Incidents

Stroke was the primary focus of the investigation. Incidences of stroke were assessed throughout the follow‐up period using a standardized survey, and individuals who answered affirmatively to the question “Have you received a stroke diagnosis from a physician?” were classified as stroke cases.

### 2.3 Definition and Categorization of DM

Diabetes status was categorized as follows: DM was diagnosed based on a self‐report from a healthcare professional or physician, corroborated by a blood test indicating FBG > 125 mg/dL or glycated hemoglobin > 6.4%. Meeting either of these criteria classified an individual as having DM. Prediabetic individuals exhibited HbA1c levels ranging from 5.7% to 6.4% (39–46 mmol/mol) or FBG concentrations between 100 and 125 mg/dL, while normoglycemic participants demonstrated HbA1c levels < 5.7% (< 39 mmol/mol) and FBG < 100 mg/dL. Blood samples were collected by trained phlebotomists following a standardized protocol.

### 2.4 Covariates

During the baseline assessment in 2011, trained interviewers employed questionnaires to gather data on sociodemographic status and health‐related variables, encompassing age, gender, place of residence, marital status (married and other), and educational attainment (less than primary school, primary school, secondary school, and higher than secondary school), as well as exercise level (none, light, moderate, and heavy). Health‐related factors encompass self‐reported smoking (past and current) and alcohol consumption habits (past and current) alongside physician‐diagnosed medical conditions such as hypertension, heart disease, liver disease, kidney disease, and dyslipidemia.

Laboratory assessments included FBG, triglycerides (TG), total cholesterol (TC), high‐density lipoprotein cholesterol (HDL‐C), low‐density lipoprotein cholesterol (LDL‐C), estimated glomerular filtration rate (eGFR), and HbA1c. The eGFR was computed using the updated CKD‐EPI equation by Inker et al. in 2021, designed to mitigate racial bias.

### 2.5 Mendelian Randomization

Drawing upon data from a previously conducted genome‐wide association analysis study (GWAS), we conducted a two‐sample MR investigation to elucidate the relationship between diabetes and stroke. To heighten the MR's sensitivity, we meticulously enforced criteria ensuring that confidence intervals (CIs) in unity retention plots did not intersect 0, thereby necessitating the exclusion of certain non‐compliant two‐sample studies. Horizontal pleiotropy in causal relationships was assessed using both the global MR‐PRESSO test and the intercept of MR‐Egger regression. Meta‐analyses of these two outcomes were performed to unveil more robust causal associations. All ethical approvals and participant consent procedures were executed in alignment with the GWAS studies from which the data for this MR investigation were derived. No further ethical review was deemed necessary.

### 2.6 Statistical Analysis

Continuous variables are expressed as means ± standard deviation, while categorical variables are presented as percentages. Initially, baseline characteristics were summarized based on diabetes status, and differences among diabetic, prediabetic, and normoglycemic participants were assessed using chi‐square tests for categorical variables and one‐way ANOVA or Wilcoxon tests for continuous variables. Subsequently, single‐factor and multifactor logistic regression analyses were conducted to estimate potential associations of gender, age, smoking, alcohol consumption, heart disease, dyslipidemia, and exercise with stroke in cohort analyses at various follow‐up intervals.

Multivariate logistic regression analyses were performed, adjusting for gender, age, smoking, alcohol consumption, heart disease, systolic blood pressure (SBP), chronic kidney disease, HDL‐C, and LDL‐C to examine the relationship between diabetes and stroke. Restricted cubic spline curves were employed to assess potential nonlinear relationships between continuous glycated hemoglobin and FBG with stroke. In addition, subgroup analyses were conducted to evaluate the impact of gender, age, marital status, place of residence, smoking, alcohol consumption, hypertension, hypertension medication, and diabetes medication on stroke outcomes. For missing data, we employed multiple imputations or deletions as appropriate.

## 3 Results

### 3.1 Baseline Characteristics of Study Participants

This study comprised 6801 middle‐aged and elderly adults from China. At baseline in 2011, the mean age was 58.43 ± 8.39 years, with 44.92% male and 55.08% female. Among these participants, 2756 (40.5%) had normoglycemia, 3008 (44.2%) had prediabetes, and 1037 (15.3%) had diabetes (Table 1). Relative to individuals with normoglycemia, those with prediabetes and diabetes tended to be older, more likely to reside in urban areas, and exhibited a higher prevalence of hypertension and heart disease. In addition, levels of FBG, TC, TG, and HbA1c were elevated, while eGFR and LDL‐C levels were diminished in these groups.

**TABLE 1:** Baseline characteristics of participants.

Variable	Overall	Missing (%)	Control	Prediabetes	Diabetes	*p*
Total participant	6801		2756	3008	1037	
Age, mean ± SD	58.433 ± 8.398	0	57.831 ± 8.544	58.756 ± 8.265	59.097 ± 8.296	< 0.001
Gender (%)						
Female	3746 (55.08)	0	1529 (55.48)	1656 (55.05)	561 (54.10)	0.747
Male	3055 (44.92)		1227 (44.52)	1352 (44.95)	476 (45.90)	
Marry (%)						
Other	671 (9.87)	0	249 (9.03)	325 (10.80)	97 (9.35)	0.066
Married	6130 (90.13)		2507 (90.97)	2683 (89.20)	940 (90.65)	
Rural (%)						
Urban	2273 (33.42)	0	864 (31.35)	1013 (33.68)	396 (38.19)	< 0.001
Rural	4528 (66.58)		1892 (68.65)	1995 (66.32)	641 (61.81)	
Ever drinking (%)	2631 (38.72)	0.1	1040 (37.78)	1173 (39.02)	418 (40.35)	0.316
Ever smoking (%)	2562 (37.68)	0	1076 (39.04)	1094 (36.38)	392 (37.80)	0.114
Hypertension (%)	1711 (25.25)	0.4	557 (20.32)	766 (25.55)	388 (37.45)	< 0.001
Heart problem (%)	754 (11.12)	0.3	259 (9.42)	341 (11.37)	154 (14.92)	< 0.001
Liver disease (%)	225 (3.32)	0.4	93 (3.38)	90 (3.01)	42 (4.07)	0.249
Kidney disease (%)	363 (5.36)	0.4	150 (5.47)	158 (5.27)	55 (5.31)	0.946
FBG, mean ± SD	108.696 ± 32.126	0	91.739 ± 6.899	107.95 ± 6.935	155.923 ± 59.238	< 0.001
TC, mean ± SD	194.388 ± 38.521	0	187.452 ± 34.641	198.055 ± 38.499	202.189 ± 44.931	< 0.001
TG, mean ± SD	131.086 ± 106.957	0	106.435 ± 54.232	131.5 ± 86.606	195.404 ± 199.343	< 0.001
HDL, mean ± SD	51.403 ± 15.222	0	53.071 ± 14.363	51.602 ± 15.45	46.391 ± 15.713	< 0.001
LDL, mean ± SD	117.612 ± 34.413	0.2	114.787 ± 31.109	120.79 ± 35.165	115.886 ± 39.507	< 0.001
HbA1, mean ± SD	5.271 ± 0.78	0	5.017 ± 0.327	5.204 ± 0.407	6.138 ± 1.516	< 0.001
eGFR, mean ± SD	96.674 ± 13.261	0	97.586 ± 12.943	96.422 ± 12.917	94.974 ± 14.809	< 0.001

*Note*: Categorical variables were expressed as counts (percentages) and compared by chi‐square test. In the case of normal distribution, continuous variables were expressed as mean ± standard deviation (SD) and compared between multiple groups by one‐way ANOVA.

Abbreviations: eGFR, estimated glomerular filtration rate; FBG, fasting blood sugar; HbA1c, hemoglobin A1C; HDL‐C, high‐density lipoprotein cholesterol; LDL‐C, low‐density lipoprotein cholesterol; missing, ratio of missing values to total participants; TC, total cholesterol; TG, triglyceride.

### 3.2 Cohort Analysis of Long‐Term Middle‐Aged and Elderly Diabetic Patients and Stroke

At different follow‐up intervals of 2, 4, 7, and 9 years, 103, 473, and 543 incident strokes were, respectively, identified. Over the course of the cohort study spanning from 2011 to 2020, the incidence of stroke among the total population, normoglycemic group, prediabetic individuals, and those with diabetes was 7.98% (543/6258), 6.97% (192/2564), 8.01% (241/2767), and 10.61% (110/927) (Table 2).

**TABLE 2:** Descriptive statistics of stroke occurrence at different follow‐up times in diabetes.

Variable	Overall	Missing (%)	Control	Prediabetes	Diabetes	*p*
Total participant Stroke	6801		2756	3008	1037	
2011–2013 (%)						
No	6441 (99.58)	4.9	2623 (99.70)	2827 (99.54)	991 (99.40)	0.419
Yes	27 (0.42)		8 (0.30)	13 (0.46)	6 (0.60)	
2011–2015 (%)						
No	6698 (98.49)	0.0	2721 (98.73)	2963 (98.50)	1014 (97.78)	0.103
Yes	103 (1.51)		35 (1.27)	45 (1.50)	23 (2.22)	
2011–2018 (%)						
No	6328 (93.05)	0.0	2591 (94.01)	2803 (93.18)	934 (90.07)	< 0.001
Yes	473 (6.95)		165 (5.99)	205 (6.82)	103 (9.93)	
2011–2020 (%)						
No	6258 (92.02)	0.0	2564 (93.03)	2767 (91.99)	927 (89.39)	0.001
Yes	543 (7.98)		192 (6.97)	241 (8.01)	110 (10.61)	

*Note*: Categorical variables were expressed as counts (percentages) and compared by chi‐square test.

Abbreviation: missing, ratio of missing values to total participants.

Initially, we conducted univariate logistic regression analyses (Table 3) across various follow‐up durations. Individuals engaging in vigorous exercise exhibited a 52% lower likelihood of experiencing a stroke event compared to non‐exercisers over a follow‐up period of up to 9 years (OR = 0.48; 95% CI: 0.30–0.78), while those engaging in heavy exercise had a reduced stroke risk over a follow‐up period of up to 4 years (OR = 0.33; 95% CI: 0.13–0.82). Moreover, risk factors such as DM, kidney disease, hypertension, dyslipidemia, smoking, and alcohol consumption were associated with an increased risk of stroke.

**TABLE 3:** Single‐factor logistic regression analysis affecting stroke occurrence.

Variables	2011–2015 (Cohort study)		2011–2020 (Cohort study)	
	*p*	OR (95% CI)	*p*	OR (95% CI)
Age	0.27	1.01 (0.99–1.04)	< 0.001	1.03 (1.02–1.04)
Gender				
Female		1.00 (Reference)		1.00 (Reference)
Male	0.001	1.95 (1.31–2.91)	0.524	1.06 (0.89–1.26)
Hypertension				
No		1.00 (Reference)		1.00 (Reference)
Yes	< 0.001	2.24 (1.51–3.32)	< 0.001	2.38 (1.99–2.85)
Heart problems				
No		1.00 (Reference)		1.00 (Reference)
Yes	< 0.001	2.23 (1.39–3.60)	< 0.001	2.10 (1.67–2.63)
Dyslipidemia				
No		1.00 (Reference)		1.00 (Reference)
Yes	0.011	1.94 (1.16–3.25)	< 0.001	2.16 (1.71–2.74)
Kidney disease				
No		1.00 (Reference)		1.00 (Reference)
Yes	0.017	2.17 (1.15–4.10)	0.049	1.41 (1.01–2.00)
Ever drinking				
No		1.00 (Reference)		1.00 (Reference)
Yes	0.04	1.50 (1.02–2.22)	0.227	1.12 (0.93–1.33)
Ever smoking				
No		1.00 (Reference)		1.00 (Reference)
Yes	< 0.001	2.07 (1.40–3.07)	0.024	1.23 (1.03–1.47)
Diabetes				
None		1.00 (Reference)		1.00 (Reference)
Prediabetes	0.464	1.18 (0.76–1.84)	0.133	1.16 (0.96–1.42)
Diabetes	0.036	1.76 (1.04–3.00)	< 0.001	1.58 (1.24–2.03)
Exercise				
None		1.00 (Reference)		1.00 (Reference)
LE	0.292	0.61 (0.24–1.53)	0.744	0.92 (0.57–1.50)
ME	0.135	0.51 (0.21–1.24)	0.36	0.80 (0.50–1.28)
HE	0.017	0.33 (0.13–0.82)	0.003	0.48 (0.30–0.78)

Abbreviations: LE, light exercise; ME, moderate exercise; VE, vigorous; exercise.

Multivariable logistic regression analyses revealed that individuals with heart disease and hypertension were more prone to experiencing a stroke overall during the 4‐year follow‐up period. In addition, during the overall 7‐year follow‐up, individuals with heart disease and hypertension were at a higher risk of stroke, and those with diabetes also demonstrated an increased likelihood of experiencing a stroke (Table S1). Furthermore, during the 2011–2018 follow‐up, there was a 2% rise in the risk of stroke for each additional year of disease duration among middle‐aged and elderly individuals with diabetes (Table S1).

**FIGURE 2:**
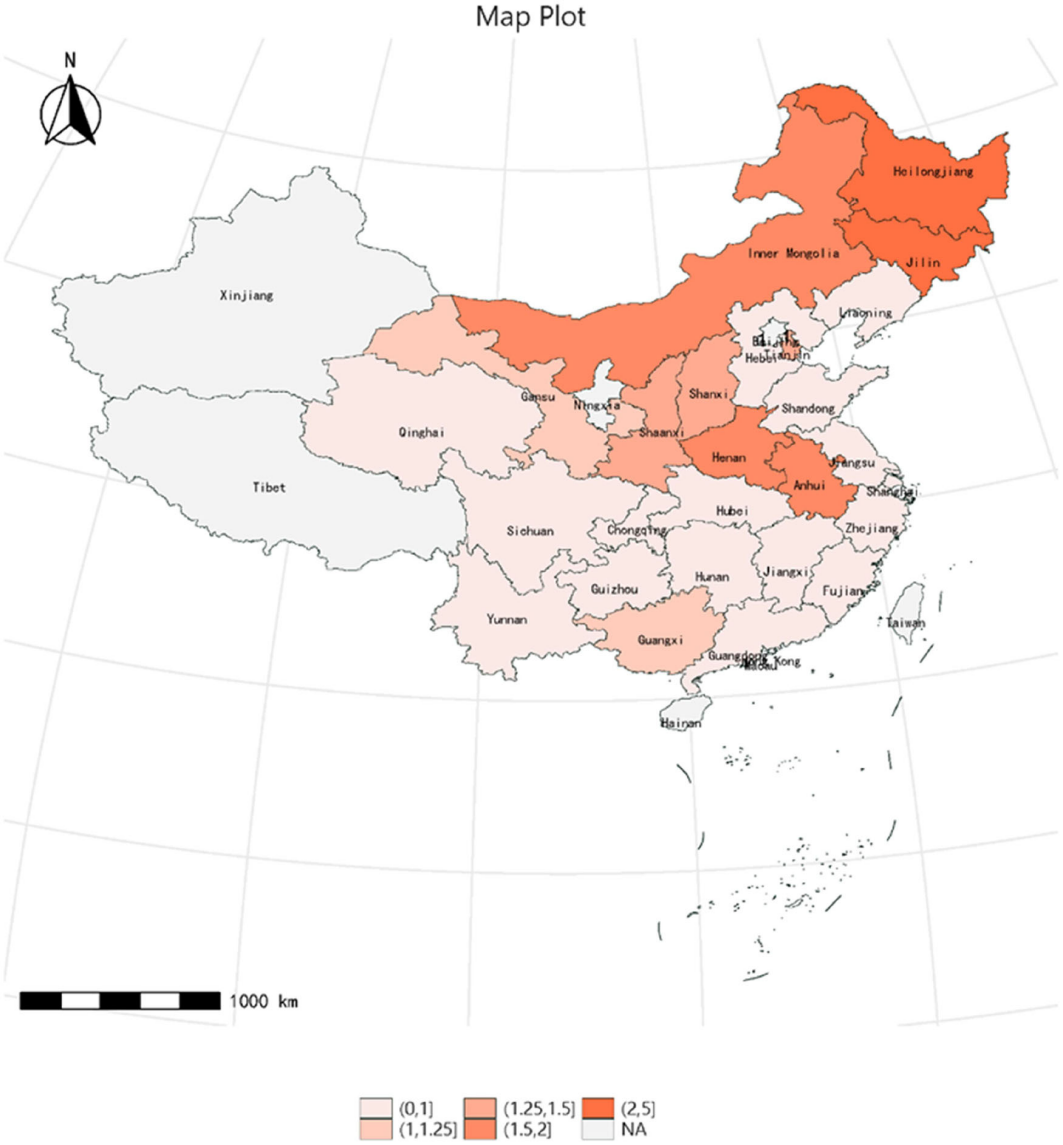
Total incidence of stroke, 2011–2020.

Furthermore, we analyzed the incidence of stroke over a 9‐year follow‐up period in both northern and southern regions of China from 2011 to 2020. Our findings revealed that the incidence of stroke was notably higher in the northern region compared to the southern region (Figure 2).

As depicted in Table 4, we conducted multimodel logistic regression analyses to investigate the association of diabetes and prediabetes with stroke over varying follow‐up durations. Our observations indicated that diabetes with longer follow‐up periods exhibited a heightened likelihood of association with stroke. Upon adjusting for potential confounders, the adjusted CIs for stroke were as follows: 95% CIs of 1.68 (1.28–2.19), 1.63 (1.25–2.13), 1.65 (1.26–2.16), and 1.35 (1.03–1.79) over the 7‐year follow‐up period. Furthermore, statistically significant differences in stroke occurrence were noted among patients with diabetes for an extended duration (more than 6 years) in multifactorial mixed model analyses.

**TABLE 4:** Association of diabetes with stroke in multiple models with different follow‐up times.

Variables	Model 1		Model 2		Model 3		Model 4	
	OR (95% CI)	*p*	OR (95% CI)	*p*	OR (95% CI)	*p*	OR (95% CI)	*p*
2011–2015								
Control	1.00 (Reference)		1.00 (Reference)		1.00 (Reference)		1.00 (Reference)	
Prediabetes	1.24 (0.78–1.95)	0.36	1.23 (0.78–1.93)	0.38	1.25 (0.79–1.97)	0.343	1.12 (0.70–1.77)	0.634
Diabetes	1.62 (0.93–2.84)	0.09	1.59 (0.91–2.79)	0.105	1.60 (0.91–2.81)	0.102	1.13 (0.63–2.03)	0.671
2011–2018								
Control	1.00 (Reference)		1.00 (Reference)		1.00 (Reference)		1.00 (Reference)	
Prediabetes	1.18 (0.95–1.46)	0.146	1.15 (0.92–1.43)	0.218	1.15 (0.92–1.43)	0.214	1.05 (0.84–1.32)	0.645
Diabetes	1.68 (1.28–2.19)	< 0.001	1.63 (1.25–2.13)	< 0.001	1.65 (1.26–2.16)	< 0.001	1.35 (1.03–1.79)	0.031
2011–2020								
Control	1.00 (Reference)		1.00 (Reference)		1.00 (Reference)		1.00 (Reference)	
Prediabetes	1.18 (0.97–1.45)	0.102	1.16 (0.94–1.42)	0.163	1.16 (0.95–1.42)	0.147	1.07 (0.87–1.32)	0.501
Diabetes	1.53 (1.19–1.98)	0.001	1.49 (1.15–1.92)	0.002	1.51 (1.17–1.95)	0.002	1.25 (0.96–1.63)	0.093

*Note*: Model 1: Crude. Model 2: Adjusted for age and gender. Model 3: Adjusted for age, gender, marital status, area, kidney disease, ever drinking, ever smoking, and education. Model 4: Adjusted for age, high‐density lipoprotein cholesterol, low‐density lipoprotein cholesterol, gender, marital status, area, kidney disease, ever drinking, ever smoking, SBP, and heart disease.

Abbreviations: CI, confidence interval; OR, odds ratio.

### 3.3 Results of Subgroup Analyses of Diabetes

**FIGURE 3:**
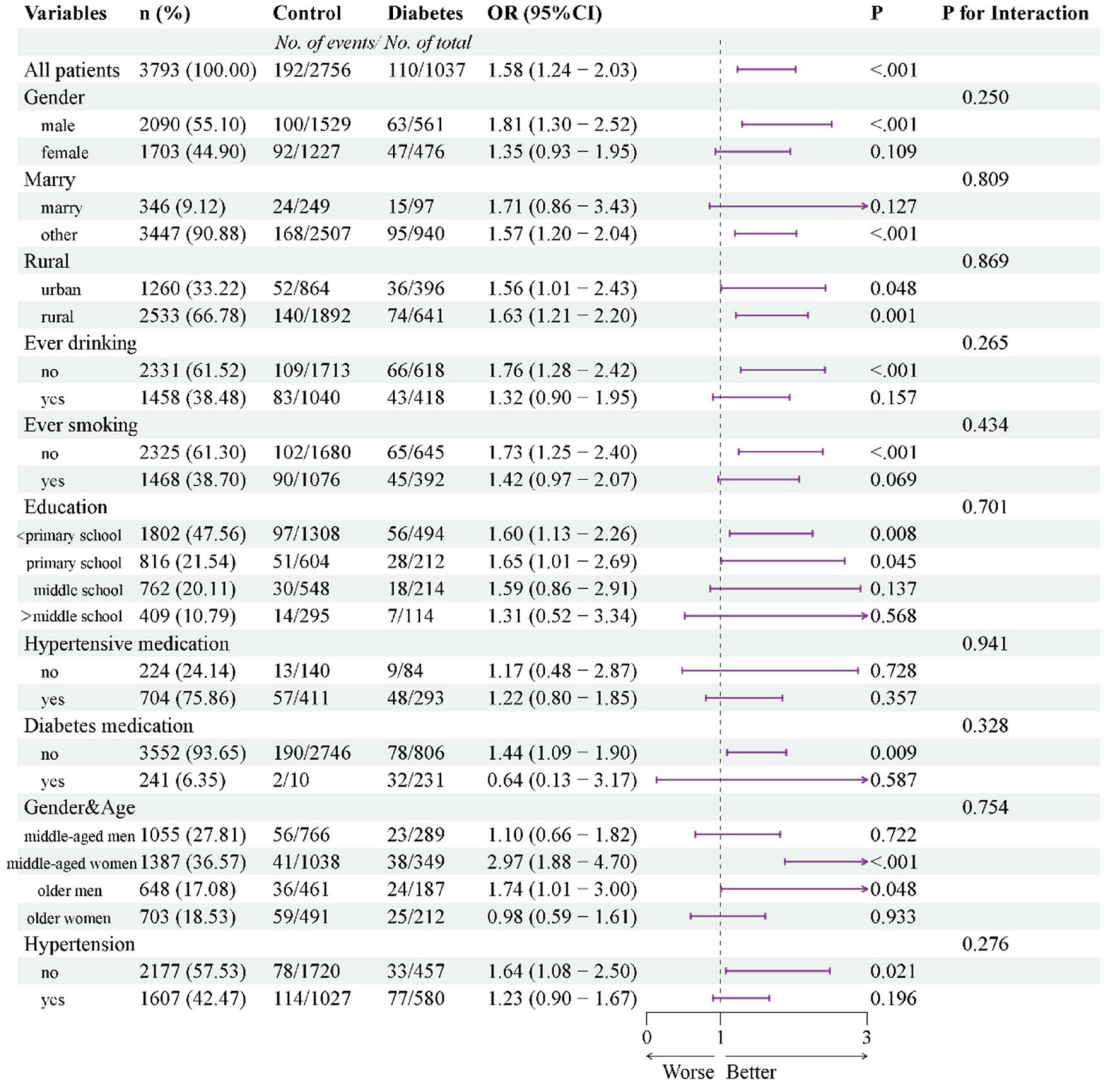
Subgroup analysis of the association between diabetes and incident stroke, 2011–2020.

Subgroup analyses were conducted to stratify the relationship between diabetes and stroke, as depicted in Figure 3. Interactions were scrutinized concerning sex, age, marital status, educational level, smoking status, alcohol consumption status, hypertension, hypertension medication, and diabetes medication. Notably, no significant interactions were detected for the association between diabetes and incident stroke across these variables (*p* > 0.05 for all interactions).

These findings suggest that the inverse association between DM and incident stroke remains consistent across diverse populations with varying demographic, socioeconomic, and health status characteristics, thereby potentially extending to broader population groups. Furthermore, subgroup analyses conducted during the follow‐up periods of 2011–2015 and 2011–2018 revealed no differences in stratification during the shorter follow‐up period (4 years) (Figures S1 and S2).

**FIGURE 4:**
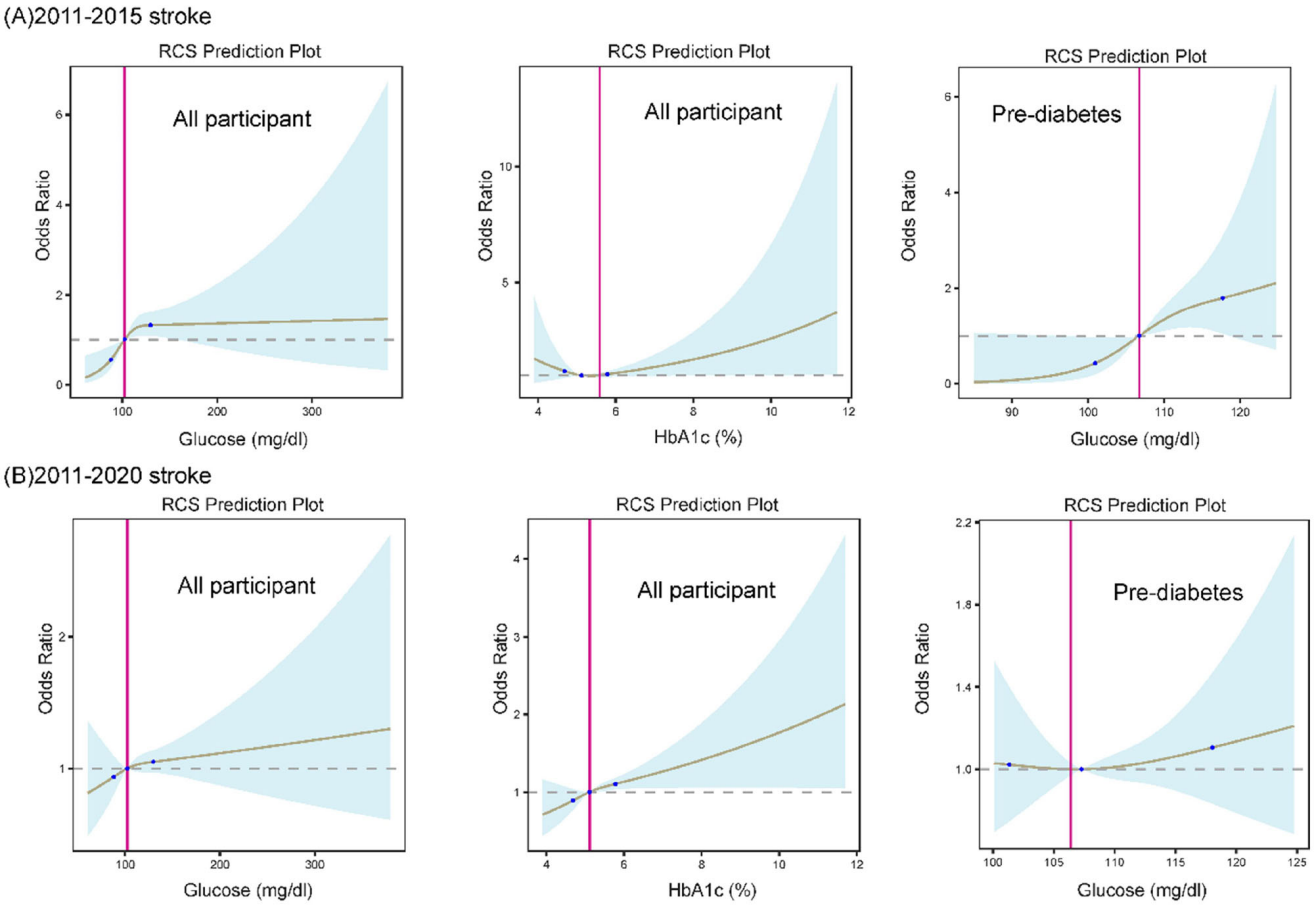
The restricted cubic spline describes the relationship between fasting glucose and glycated hemoglobin with the risk of stroke. (A) Fasting glucose and HbA1c versus dose‐effect analysis for participants at risk of stroke 2011–2015. (B) Fasting glucose and glycated haemoglobin versus dose‐effect analysis for participants at risk of stroke 2011–2020. The grey shaded area around the restricted cubic spline curve in the figure indicates the 95% confidence interval.

After adjusting for relevant confounders, a positive dose–response relationship (nonlinear, *p* > 0.05) was observed between HbA1c concentration exceeding 5.1% and stroke risk in the total middle‐aged and elderly population from 2011 to 2020 (Figure 4B). Similarly, a comparable positive dose–response relationship (nonlinear, *p* > 0.05) was found between HbA1c concentration exceeding 5.5% and stroke risk from 2011 to 2015 (Figure 4A). Fasting glucose concentration in patients with prediabetes exhibited a positive dose–response association with stroke risk from 2011 to 2015.

In univariate regression, diabetes patients appeared to have an association with stroke occurrence. However, no positive dose–response relationship was evident between fasting glucose concentration in diabetes patients and stroke risk in the restricted cubic spline curve (Figure S3).

### 3.4 MR Analysis

**FIGURE 5:**
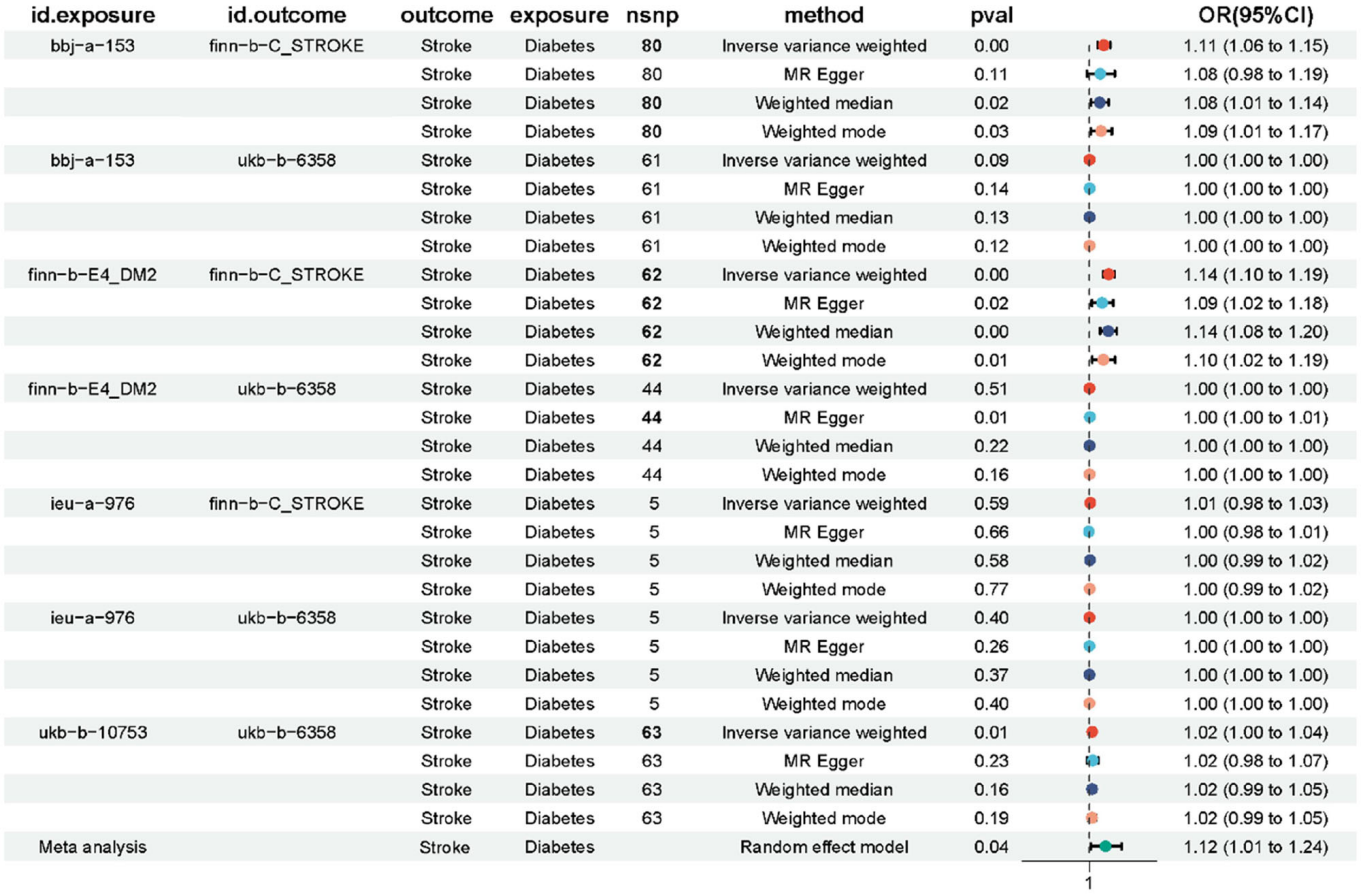
A causal association between diabetes and stroke based on Mendelian randomization.

Furthermore, we employed MR to explore the association between diabetes and stroke. Diabetes exposure data were sourced from four distinct databases, including BBJ, Finn for Type 2 diabetes, and the UKB database for physician‐diagnosed diabetes, with stroke as the outcome. Meta‐analysis unveiled a causal effect of diabetes on the incidence of stroke (Figure 5). Through leave‐one‐out analysis, the results remained consistent even after excluding each SNP individually, underscoring the reliability of our study findings (Figure S4). Moreover, heterogeneity tests and multivariate analyses demonstrated the robustness of the causal relationship between the observed associations (Tables S2 and S3).

### 3.5 Mediation Analysis

**FIGURE 6:**
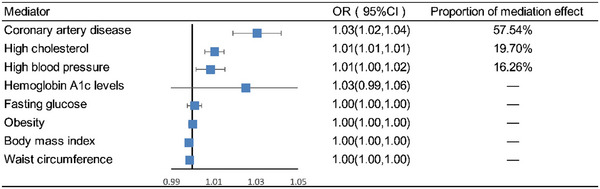
Genetically estimated mediation pathways of diabetes leading to stroke.

Coronary artery disease (mediation effect = 0.575, 95% CI: 1.019–1.042, mediated proportion = 57.54%), high cholesterol (mediation effect = 0.197, 95% CI: 1.006–1.015, mediated proportion  = 19.70%), and high blood pressure (mediation effect = 0.163, 95% CI: 1.002–1.016, mediated proportion = 16.26%) were identified as major mediators in how diabetes leads to stroke (Figure 6).

## 4 Discussion

Our research unveiled that among middle‐aged and older individuals aged 45 years and above in the CHARLS national dataset, the likelihood of experiencing a stroke was 1.35 times greater in participants with diabetes compared to those with normal blood sugar levels over a 7‐year follow‐up period. These results were corroborated by a previous meta‐analysis indicating an ischemic stroke risk ratio of 2.3 (95% CI: 2.0–2.7) in diabetic versus nondiabetic patients (The Emerging Risk Factors Collaboration 2010). In a prospective study involving 13,129 men and 22,528 women aged 40–69 years, Cui et al. discovered that diabetes posed a risk for ischemic stroke among middle‐aged Japanese individuals, with the risk being two to three times higher than that of individuals with normal blood sugar levels (Cui et al. 2011). The observational study by Stratton et al. found that in patients with Type 2 diabetes, the risk of diabetes complications is closely related to prior high blood glucose levels (Klatsky, Armstrong, and Friedman 1990). However, our study differed from others in two main aspects: first, we focused on examining a nationally representative group of middle‐aged and elderly adults aged 45 and above in China, and second, our research centered on conducting a cohort analysis of newly occurring strokes throughout the nine‐year follow‐up duration.

In our investigation, we initially identified that among middle‐aged and elderly individuals with prediabetes, having blood glucose levels above 106.7 mg/dL posed a risk factor for stroke, and this risk escalated with higher FBG concentrations, while the Manhattan study identified this threshold at 126 mg/dL (Stratton et al. 2000). Thus, blood glucose control should be tailored based on regional, dietary, and physiological differences. However, when the follow‐up duration exceeded 7 years, there was no discernible trend indicating a linear association between fasting glucose levels above 106.7 mg/dL and stroke risk. Jin et al. conducted a study involving 96,110 participants, discovering that FBG levels ranging from 6.1 to 6.9 mmol/L, indicative of impaired fasting glucose, were linked to an elevated risk of intracranial hemorrhage (Boden‐Albala et al. 2008). Previous studies have reported that prediabetes is associated with an increased risk of stroke only in nonhypertensive populations (Katan and Luft 2018). Lee et al. analyzed 15 prospective cohort studies involving 760,925 participants. Among these, eight studies focused on impaired glucose tolerance and impaired fasting glucose. They revealed a 1.26‐fold increased risk of stroke (95% CI: 1.10–1.43) after adjusting for recognized cardiovascular risk factors (Iwahana et al. 2011). Lee et al. proposed that the existing definition of impaired fasting glucose (100–125 mg/dL) should be revised to 110–125 mg/dL to more accurately indicate the heightened sub‐risk of stroke. Our study provides additional support for this perspective.

The literature on the association between diabetes and stroke outcomes presents inconsistencies and variations (Fang et al. 2005; Jin et al. 2018). Therefore, we not only used fasting glucose to evaluate the risk of stroke but also adopted HbA1c as a diagnostic tool for DM to better assess the long‐term effects of glucose. A glycated hemoglobin concentration exceeding 5.1% emerged as a risk factor for stroke in all Chinese middle‐aged and elderly participants during a 9‐year follow‐up period, with stroke risk escalating with elevated glycated hemoglobin levels. Stergaard et al. reported that increased hemoglobin glycation indices were linked to elevated cardiovascular event risk in Type 2 DM patients without prior cardiovascular disease (Wang et al. 2022). Meta‐analyses and sensitivity analyses conducted by Mitsios et al. involved 532,779 participants from 29 studies. Their findings revealed that with each 1% increase in HbA1c levels, the risk of first‐ever ischemic stroke rose in both nondiabetic and diabetic cohorts (Lee et al. 2012).

DM emerged as a prominent risk factor for stroke across all clinically significant subgroups we analyzed. However, the odds ratio exhibited notably higher significance in certain demographics with comparatively lower inherent risk of vascular disease, particularly among middle‐aged women, nonsmokers, and nondrinkers. In light‐drinking populations, lower cardiovascular events may be due to its role in preventing atherosclerosis or abstinence from alcohol due to noncardiovascular symptoms in drinking populations (Elley et al. 2008). In a meta‐analysis of 102 prospective studies, it was observed that the hazard ratio for ischemic stroke tended to be greater among women compared to men, likely attributed to the higher prevalence of hypertension and atrial fibrillation, the primary stroke risk factors in the female population (The Emerging Risk Factors Collaboration 2010; Lau et al. 2019; Østergaard et al. 2019; Mitsios et al. 2018). Our study found that intensive physical activities (e.g., aerobic exercise, plowing) reduced the risk of stroke, whereas light to moderate exercise (e.g., walking, mopping the floor, and Tai Chi) did not decrease the risk of stroke. Willey et al. recruited 3298 participants and found that in a prospective cohort, moderate to heavy physical activity, without energy expenditure, prevented the risk of ischemic stroke independently of other stroke risk factors (Willey et al. 2009). Therefore, middle‐aged and elderly individuals should be encouraged to engage in appropriate‐intensity physical activities to reduce the risk of new strokes (Wendel‐Vos 2004). The incidence of stroke is higher in northern China than in southern China, possibly due to the influence of traditional northern dietary habits, characterized by higher consumption of refined grain products and pickled vegetables, which are associated with an increased risk of stroke (Li et al. 2011). The duration of diabetes was also associated with an elevated risk of stroke, with a 2% increase in risk observed for each additional year of diabetes duration. In addition, it is worth noting that the shorter 3‐year follow‐up did not see a difference in stroke risk.

We conducted an additional meta‐analysis of GWAS data sourced from various databases, which revealed that diabetes could potentially contribute to stroke. Employing an MR approach, we delved into the genetic perspective to explore the causal relationship between diabetes and stroke. Given that Type 2 diabetes predominates among middle‐aged and older adults, we specifically analyzed the causal link between Type 2 diabetes and stroke. Our findings suggested that Type 2 diabetes might indeed act as a risk factor for stroke. Subsequent meta‐analyses further affirmed a robust causal association between Type 2 diabetes and stroke, thereby underscoring diabetes as a significant risk factor for stroke occurrence. Previous studies have shown that individuals with diabetes have higher SBP, TC, BMI, and waist circumference compared to those without diabetes (Peters, Huxley, and Woodward 2014). In our study, we found that hypertension and high cholesterol might act as mediators in the occurrence of stroke caused by diabetes, whereas blood glucose levels did not play a mediating role in the development of stroke due to diabetes.

It is crucial to acknowledge the limitations of this study. First, while we adjusted for a set of potential confounders based on prior research, there were still some factors, such as diet, that were not included. Second, the sample size of our study was insufficient, and certain important variables from the CHARLS database, including detailed information on stroke subtypes, could not be fully incorporated, limiting our ability to conduct deeper statistical analyses.

## 5 Conclusion

In conclusion, our cohort study and MR analysis offer compelling evidence that DM is associated with an increased risk of stroke among middle‐aged and elderly Chinese individuals. In addition, elevated prediabetic blood glucose levels are positively correlated with the occurrence of stroke. Controlling glycated hemoglobin concentrations and engaging in vigorous exercise may contribute to reducing the incidence of stroke.

## Author Contributions

**Zetai Bai**: conceptualization, data curation, formal analysis, visualization, writing–original draft. **Zheyi Wang**: methodology, software, validation, writing–review and editing, writing–original draft. **Mei Li**: conceptualization, data curation, formal analysis, methodology, supervision, writing–review and editing. **Deyuan Kong**: conceptualization, methodology, software, data curation, formal analysis, visualization, writing–original draft, writing–review and editing. **Guanzhao Wu**: investigation, supervision, project administration, writing–review and editing, resources, funding acquisition, validation, software, methodology.

## Ethics Statement

The study was approved by the ethics review committee (institutional review board) of Peking University. All participants granted formal written consent for their involvement. All data are openly published as microdata at http://opendata.pku.edu.cn/dataverse/CHARLS with no direct contact with all participants.

## Conflicts of Interest

The authors declare no conflicts of interest.

### Peer Review

The peer review history for this article is available at https://publons.com/publon/10.1002/brb3.70151.

## Supporting information



Table S1 Multiple logistic regression to analyze the association of different factors with stroke risk.
Figure S1 Subgroup analysis of the association between diabetes and incident stroke, 2011‐2018.
Figure S2 Subgroup analysis of the association between diabetes and incident stroke, 2011‐2015.
Figure S3 The restricted cubic spline for the association of fasting blood glucose and stroke risk.
Figure S4 leave‐one‐out analysis

Table S2

Table S3

## Data Availability

The data that support the findings of this study are available from the corresponding author upon reasonable request.
