# Supplementary material for: Diabetes Mellitus and Risk of Future Stroke: Evidence From CHARLS and Mendelian Randomization Analyses
Source: Brain Behav. 2024 Nov 17;14(11):e70151. doi: 10.1002/brb3.70151 (PMC11570678; doi:10.1002/brb3.70151)

# Supplement materials

Table S1 Multiple logistic regression to analyze the association of different factors with stroke risk.

| Variables | 2011-2018(stroke) | |  | 2011-2015(stroke) | |
| --- | --- | --- | --- | --- | --- |
|  | *P* | OR (95%CI) |  | *P* | OR (95%CI) |
| Age | **0.001** | 1.02 (1.01 ~ 1.03) |  | 0.708 | 1.00 (0.98 ~ 1.03) |
| HDL | **<.001** | 0.99 (0.98 ~ 0.99) |  | **<.001** | 0.97 (0.96 ~ 0.99) |
| LDL | 0.072 | 1.00 (1.00 ~ 1.01) |  | 0.807 | 1.00 (0.99 ~ 1.00) |
| Gender |  |  |  |  |  |
| female |  | 1.00 (Reference) |  |  | 1.00 (Reference) |
| male | 0.138 | 0.80 (0.60 ~ 1.07) |  | 0.314 | 1.36 (0.75 ~ 2.46) |
| Rural |  |  |  |  |  |
| No |  | 1.00 (Reference) |  |  | 1.00 (Reference) |
| Yes | 0.122 | 1.18 (0.96 ~ 1.44) |  | 0.589 | 0.89 (0.59 ~ 1.34) |
| Hearte problem |  |  |  |  |  |
| No |  | 1.00 (Reference) |  |  | 1.00 (Reference) |
| Yes | **<.001** | 1.73 (1.35 ~ 2.22) |  | **0.013** | 1.87 (1.14 ~ 3.06) |
| Ever drinking |  |  |  |  |  |
| No |  | 1.00 (Reference) |  |  | 1.00 (Reference) |
| Yes | 0.135 | 1.20 (0.95 ~ 1.51) |  | 0.836 | 1.05 (0.66 ~ 1.68) |
| Ever smoking |  |  |  |  |  |
| No |  | 1.00 (Reference) |  |  | 1.00 (Reference) |
| Yes | 0.069 | 1.29 (0.98 ~ 1.70) |  | 0.062 | 1.70 (0.97 ~ 2.96) |
| Hypertension |  |  |  |  |  |
| No |  | 1.00 (Reference) |  |  | 1.00 (Reference) |
| Yes | **<.001** | 2.28 (1.86 ~ 2.80) |  | **<.001** | 2.27 (1.48 ~ 3.47) |
| Diabetes |  |  |  |  |  |
| No |  | 1.00 (Reference) |  |  | 1.00 (Reference) |
| Pre-diabetes | 0.773 | 1.03 (0.83 ~ 1.28) |  | 0.776 | 1.07 (0.68 ~ 1.68) |
| Diabetes | **0.045** | 1.32 (1.01 ~ 1.72) |  | 0.582 | 1.17 (0.67 ~ 2.04) |

OR, odds ratio;CI: confidence.;2011-2018, Risk of Stroke Occurrence, 2011 to 2018; 2011-2015, Risk of Stroke Occurrence, 2011 to 2015;

Figure S1 Subgroup analysis of the association between diabetes and incident stroke, 2011-2018.


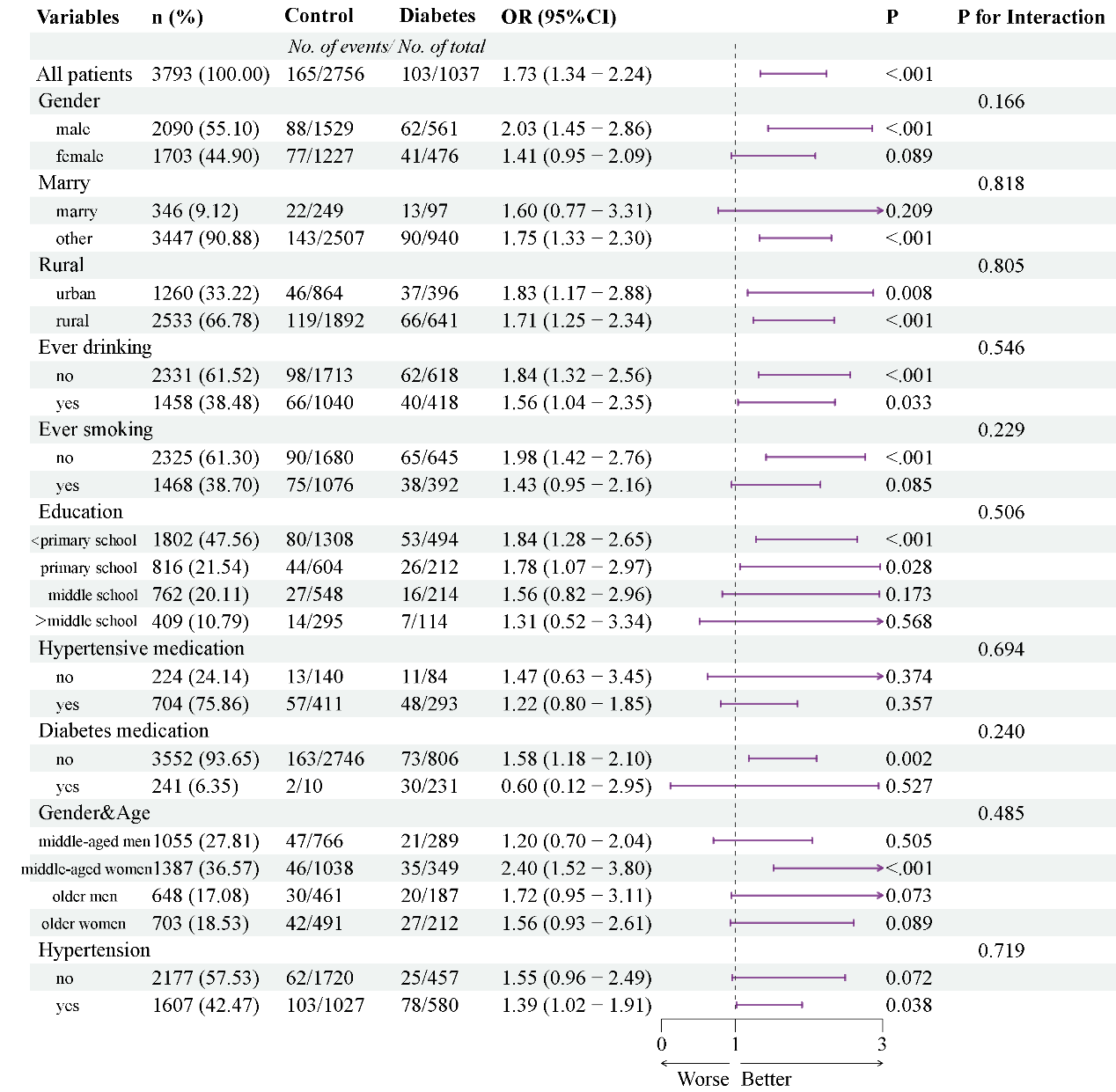


Figure S2 Subgroup analysis of the association between diabetes and incident stroke, 2011-2015.


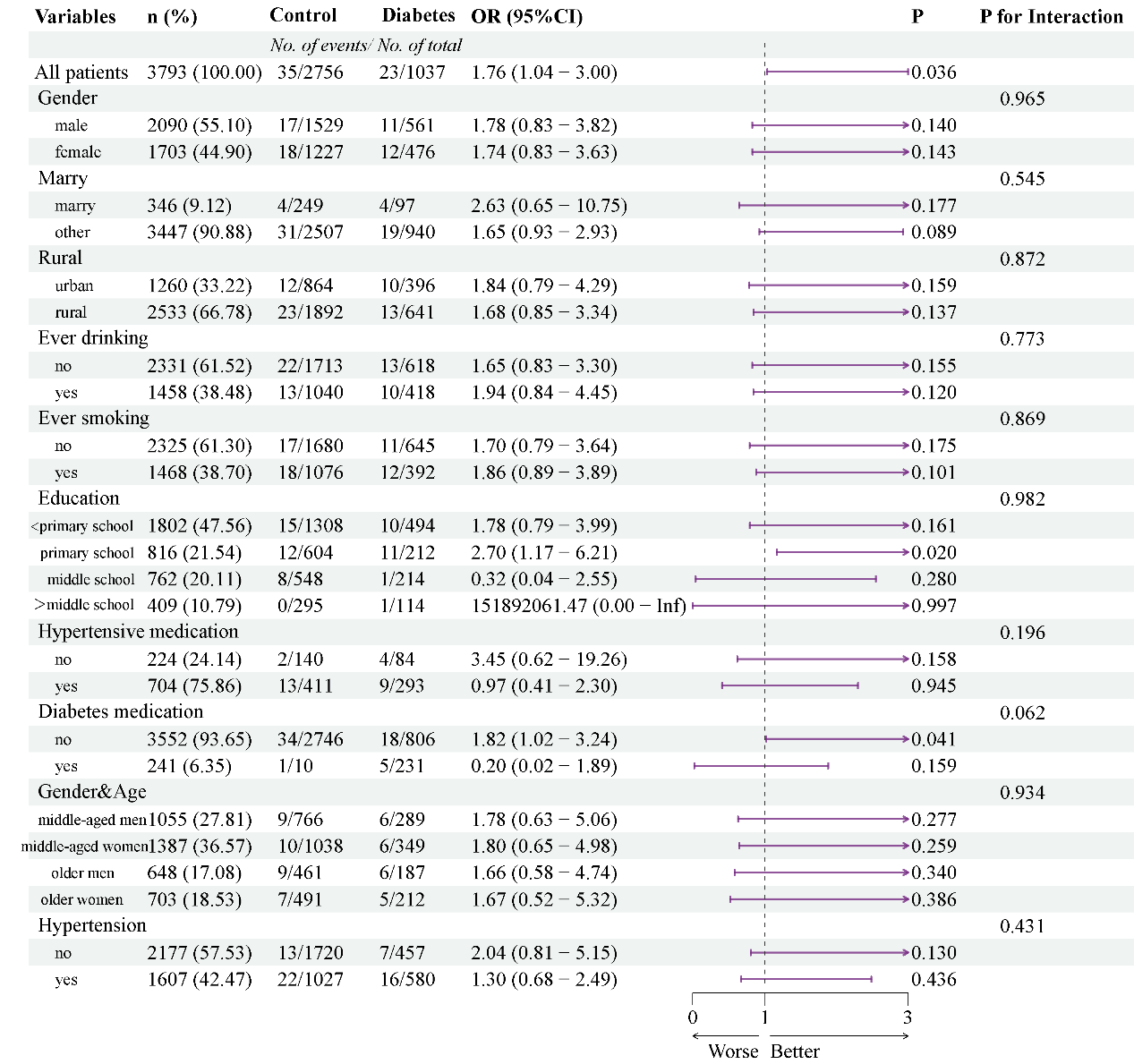


Figure S3 The restricted cubic spline for the association of fasting blood glucose and stroke risk.


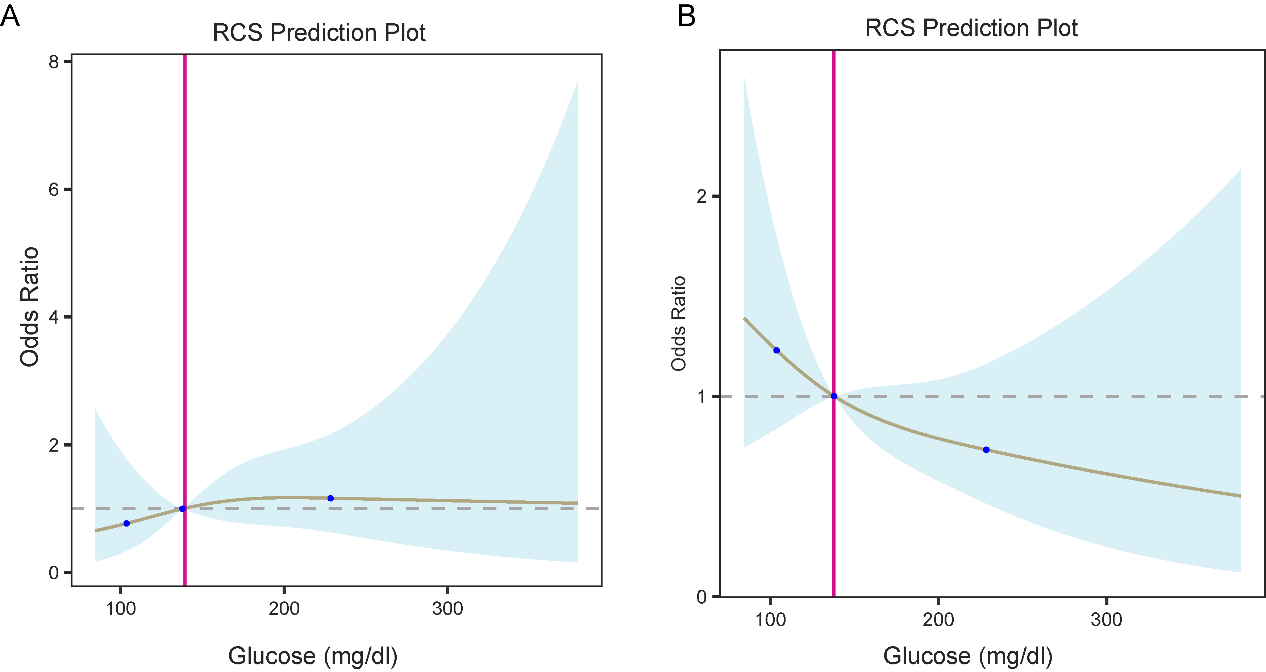


A.Restricted cubic spline of fasting glucose and stroke in middle-aged and elderly diabetic patients during the follow-up period 2011-2015 B. Restricted cubic spline of fasting glucose and stroke in middle-aged and elderly diabetic patients during the follow-up period 2011-2020

Figure S4 leave-one-out analysis


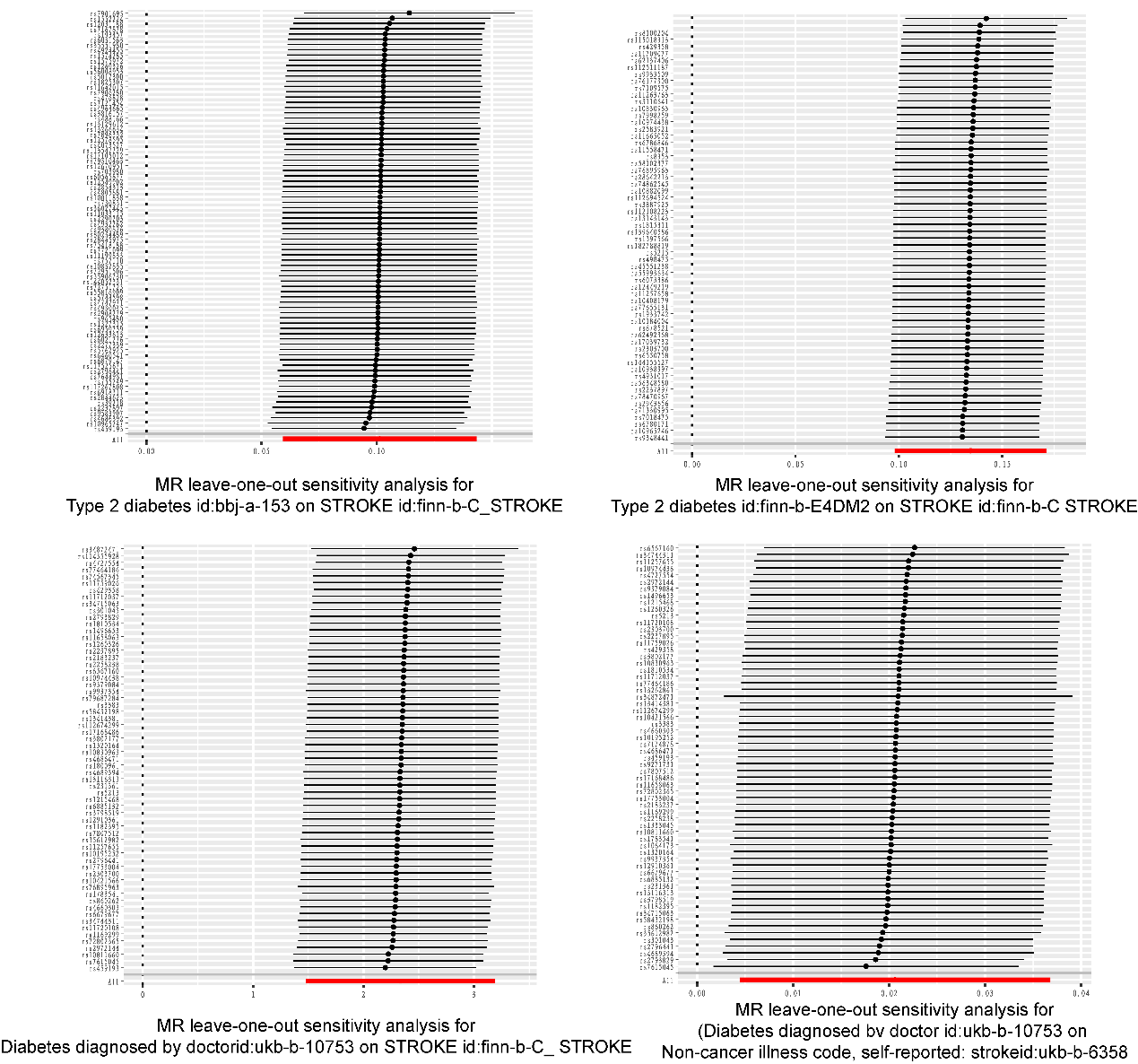

Supplement: Supplementary file 1 — Table S1 Multiple logistic regression to analyze the association of different factors with stroke risk. Figure S1 Subgroup analysis of the association between diabetes and incident stroke, 2011‐2018. Figure S2 Subgroup analysis of the association between diabetes and incident stroke, 2011‐2015. Figure S3 The restricted cubic spline for the association of fasting blood glucose and stroke risk. Figure S4 leave‐one‐out analysis [file BRB3-14-e70151-s003.docx]
